# Supplementary material for: Low circulating miR-190a-5p predicts progression of chronic kidney disease
Source: Nat Commun. 2025 Oct 15;16:9154. doi: 10.1038/s41467-025-64168-6 (PMC12528679; doi:10.1038/s41467-025-64168-6)
Supplement: Supplementary file 1 — Supplementary Information [file 41467_2025_64168_MOESM1_ESM.pdf]

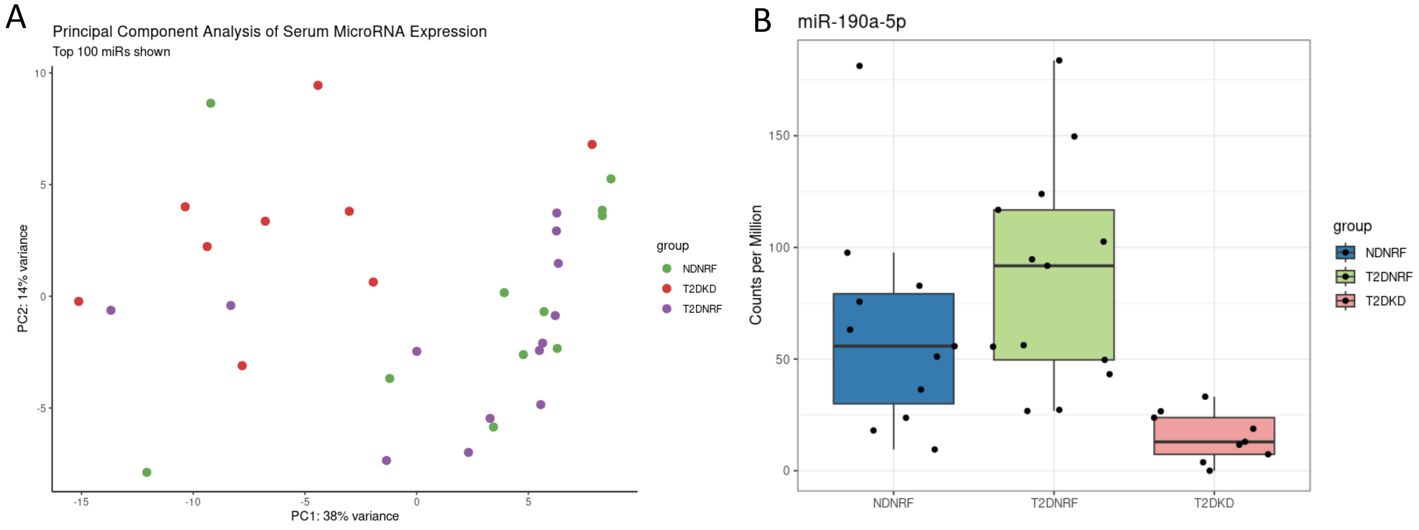

Supplementary Figure 1: Small RNA sequencing of the circulating miRNome.

A. Principal component analysis (PCA) of the top 100 most variant miRs (normalised counts) across NDNRF, T2DNRF and T2DKD samples B. Expression of miR-190a-5p normalized counts (CPM) in the discovery patient cohort. The box (IQR) is the median, Q1 (25th percentile), Q3 (75th percentile). The whiskers represent: Minimum Q1-1.5*IQR and Maximum Q3-1.5*IQR. NDNRF – Non-diabetic controls with renal function, T2DNF – Type 2 Diabetes with normal renal function, T2DKD - Type 2 Diabetes with reduced renal function.’

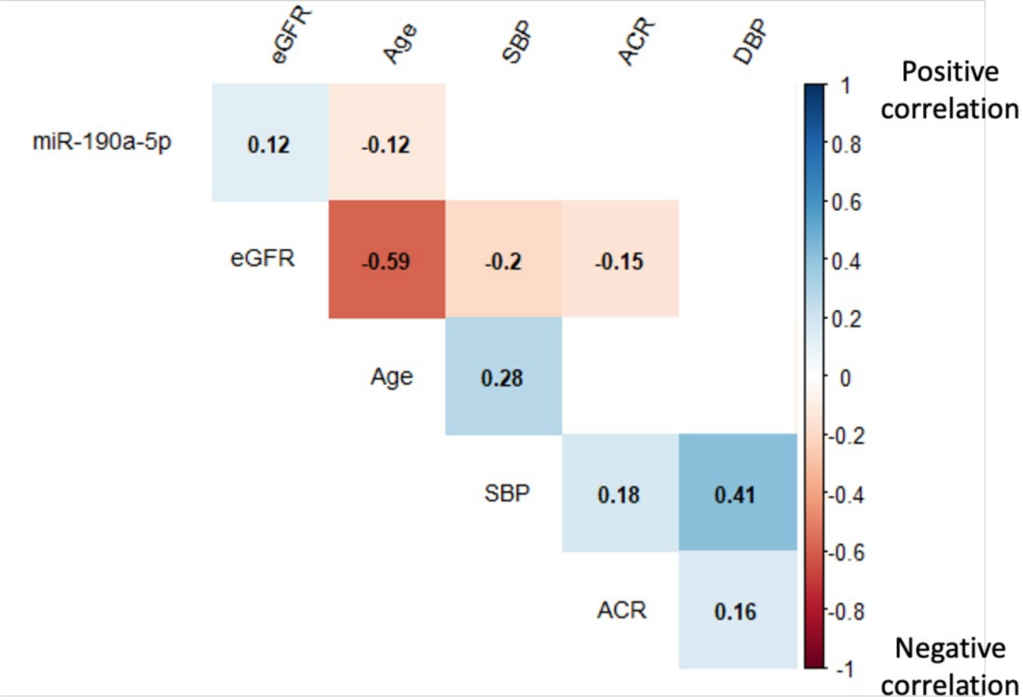

Supplementary Figure 2. Correlation matrix describing the relationships between clinical characteristics and serum miR-190a-5p level. Correlation coefficients (r) were estimated using Spearman's rank tests with only those with $p<0.05$ shown in the table.

A

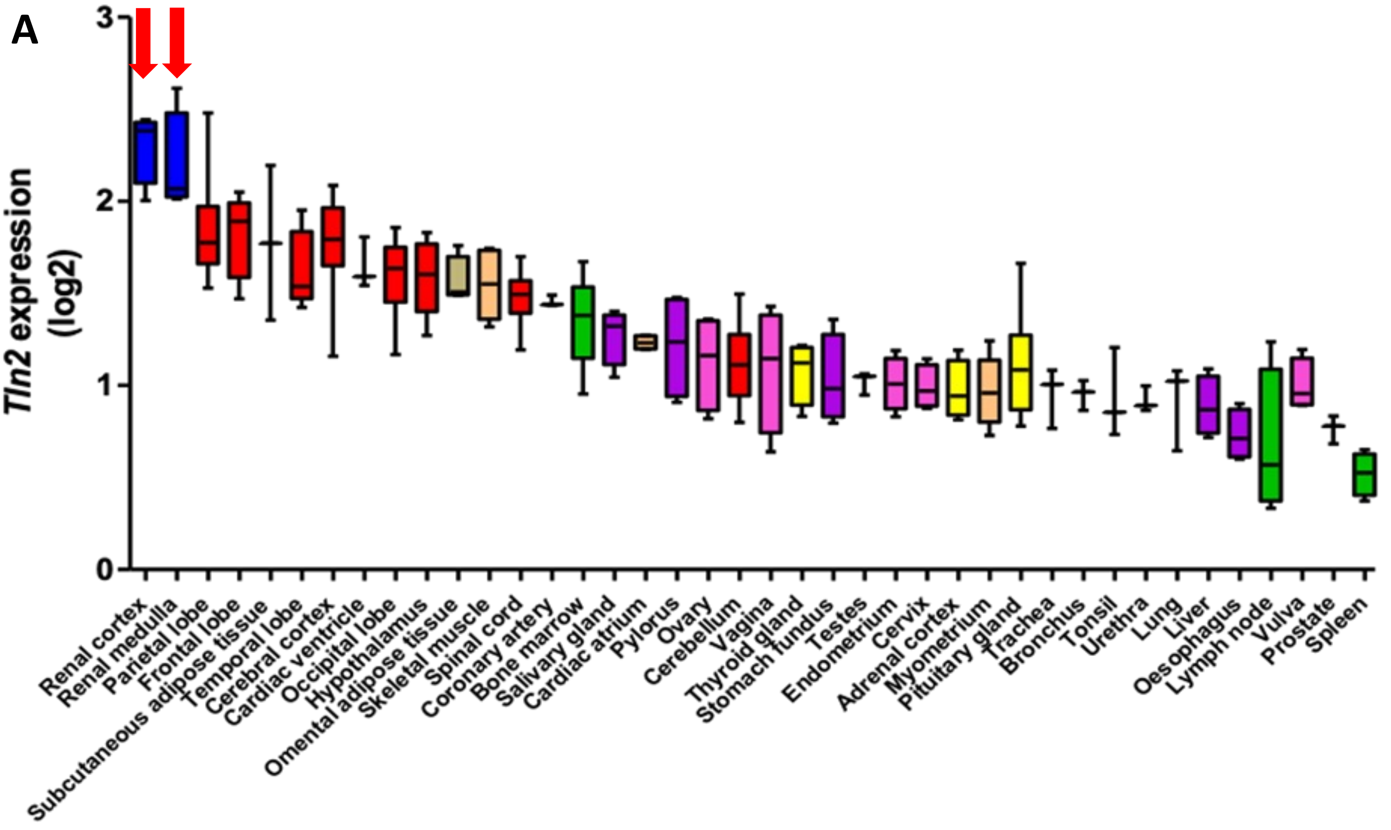

B

HUMAN	- UGAUAUGUUUGAUAUAUUAGGU *hsa-miR-190a-5p*
RAT	- UGAUAUGUUUGAUAUAUUAGGU *rno-miR-190a-5p*
MOUSE	- UGAUAUGUUUGAUAUAUUAGGU *mmu-miR-190a-5p*

C

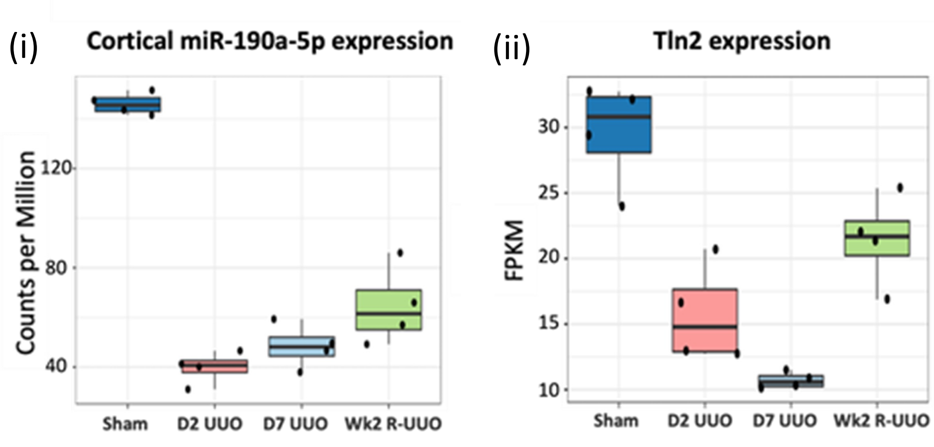

Supplementary Figure 3: Expression of miR-190a-5p and its host gene *TLN2* in human organs and pre-clinical model. A. Analysis of Tln2 in human tissue data set (GSE3526) revealing highest expression in the kidney (red arrows). B. Conservation of miR-190a-5p across species. C. Expression of miR-190a-5p (i) and Tln2 (ii) in matched bulk cortical rUUO tissue. Data expressed as box plots were the box (IQR) is the median, Q1 (25th percentile), Q3 (75th percentile). The whiskers represent: Minimum Q1-1.5*IQR and Maximum Q3-1.5*IQR.

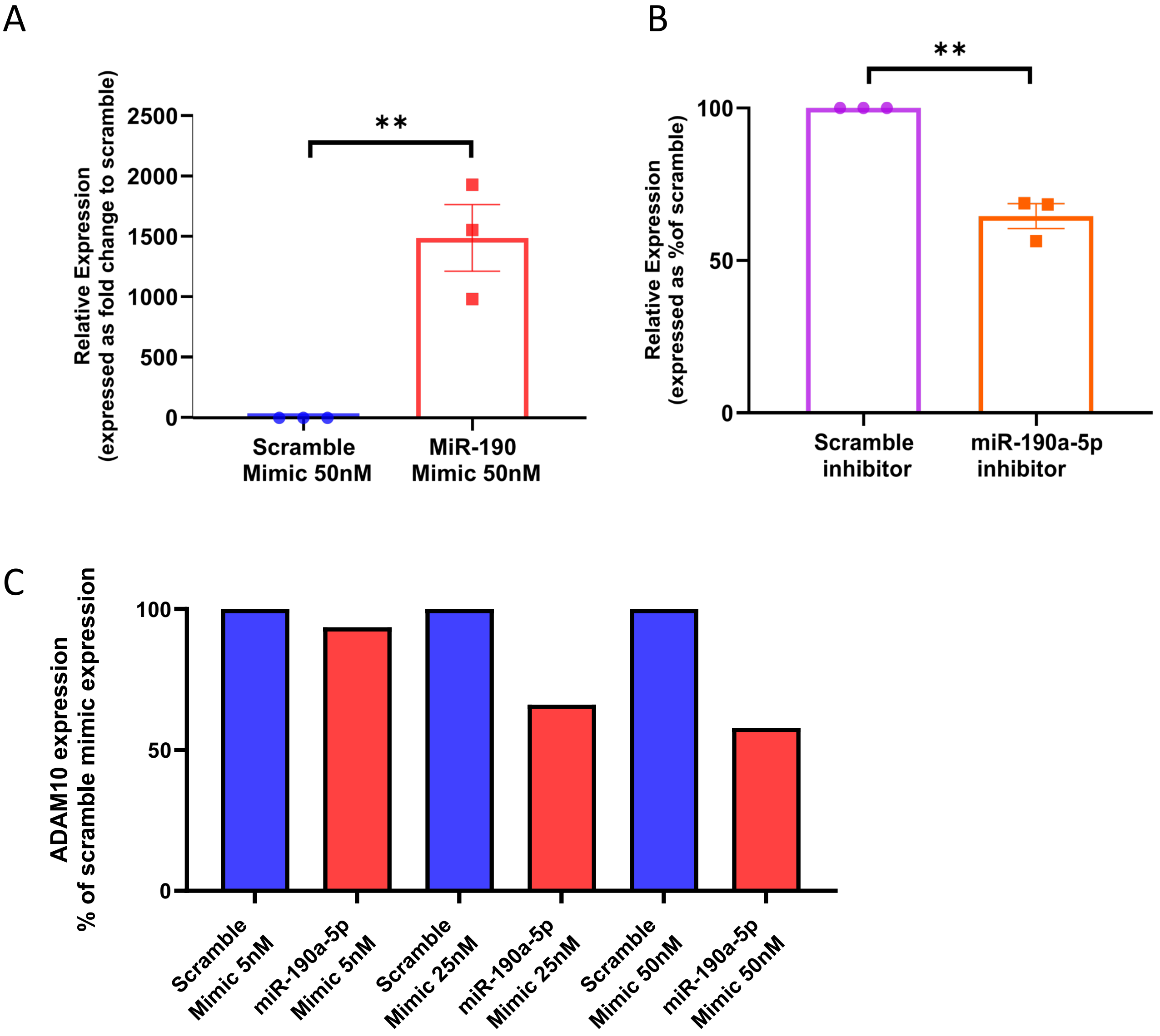

Supplementary Figure 4: miR-190a-5p modulation in RPTECS and effect on *ADAM10* expression.
Human renal proximal tubular cells were transfected with **A**. miR-190a-5p mimic (50nM)(n=3 biological repeats) or **B.** hairpin inhibitor (100nM)) or matched scrambled control (n=3 biological repeats). RNA was extracted and miR-190-5p expression measured by qPCR and normalised to RNU48. Data is expressed as fold change/percentage of scramble. Analysed by unpaired Student's T-test. **p=0.0011 (Mimic)/p=0.0034 (Inhibitor) **C.** Dose response of miR-190a-5p mimic from 5nM to 50nM on ADAM10 expression (normalised to *PPIA* or *GAPDH*) as a % of scramble control ADAM10 expression to account for off target effects.

Supplementary Tables

A

Gene	Probe/Assay ID
Adam10	Mm00545742_m1
ADAM10	Hs00153853_m1
Ass1	Mm00711256_m1
Egf	Mm00438696_m1
Col1a1	Mm00801666_g1
Col3a1	Mm00802300_m1
Ppia	Mm02342430_g1
PPIA	Hs03045993_gH
GAPDH	Hs02758991_g1

B

Target	Primer/Probe ID
Hsa-miR-190a-5p	000489
U6	001973
RNU48	001006
C.Elegans miR-39	000200

Supplementary Table 1 – Taqman Primer:Probes utilised in study. **A.** List of primers used for qPCR in human and mouse **B**. List of Primer:Probes for miRNA and snoRNA controls.

	seNSOR cohort with miR-190a-5p	Unable to record miR-190a-5p	p-value
N	298	97	
Male/Female, n	156/142	53/44	0.78
Age (years), median(IQR)	54 (41, 68)	59 (45,69)	0.11
eGFR (ml/min/1.73m^2), median(IQR)	61 (37, 93)	50 (31,79)	0.018
SBP (mmHg), median(IQR)	130 (120, 145)	128 (120,140)	0.34
DBP (mmHg), median(IQR)	78 (70, 82)	72 (68,80)	0.023
ACR (mg/mmol), median(IQR)	19 (3, 140)	27 (1,230)	0.29
ACR < 3 mg/mmol, n (%)	67 (22)	24 (25)	
ACR 3-300 mg/mmol, n (%)	156 (52)	47 (48)	
ACR >300 mg/mmol, n (%)	29 (10)	20 (21)	
ACR unavailable, n (%)	46 (15)	6 (6)	
Diabetes prevalence, n (%)	48 (16)	16 (16)	
Ethnicity			
White, n (%)	261 (88)	91 (94)	
Asian, n (%)	14 (5)	2 (2)	
Black, n (%)	6 (2)	0	
Not recorded, n (%)	17 (6)	4 (4)	
Primary kidney diagnosis			
Primary Glomerulonephritis , n (%)	85 (29)	22 (23)	
Interstitial Nephropathies, n (%)	77 (26)	28 (29)	
Multisystem diseases, n (%)	67 (22)	24 (24)	
Diabetic Nephropathy, n (%)	18 (6)	8 (8)	
Other, n (%)	14 (5)	6 (6)	
Not known, n (%)	37 (12)	9 (9)	

Supplementary Table 2. Baseline clinical characteristics of patients with and without detectable levels of miR-190a-5p in the serum. Categorical values were assessed using a Chi-square test. For continuous values without normal distribution, Mann-Whitney test was used to compare 2 groups.

	Adjusted, multivariate analysis		
	**HR**	**95% CI**	**p**
log2 miR-190a-5p	0.807	0.667-0.977	0.028
Baseline eGFR per 10ml/min/1.73m^2	0.782	0.668-0.916	0.002
ACR per 10 mg/mmol	1.040	1.001-1.082	0.046
Age per 10 years	0.777	0.587-1.029	0.079
SBP per 10 mmHg	1.138	0.963-1.345	0.129
DBP per 10 mmHg	1.005	0.743-1.360	0.975
Female (vs male)	1.263	0.472-1.180	0.529
Urinary EGF: Creatinine Ratio	0.747	0.472-1.180	0.211
Urinary KIM-1: Creatinine Ratio	1.150	0.391-3.383	0.799

Supplementary Table 3. Multi-variate Cox proportional hazards models for the secondary endpoint of progression to end-stage kidney disease in patients with baseline urinary ACR <300 mg/mmol only.

eGFR: estimated glomerular filtration rate, ACR: urinary albumin:creatinine ratio, SBP: systolic blood pressure, DBP: diastolic blood pressure

	Unadjusted, univariate analysis			*Adjusted, multivariate analysis*		
	**HR**	**95% CI**	**p**	**HR**	**95% CI**	**p**
log2 miR-190a-5p	0.89	0.77-1.02	0.087	0.86	0.73-1.01	0.064
Baseline eGFR per 10ml/min/1.73m^2	0.86	0.79-0.94	<0.001	0.82	0.73-0.92	<0.001
ACR per 10mg/mmol	1.02	1.01-1.03	<0.001	1.02	1.01-1.03	<0.001
Age per 10 years	1.05	0.91-1.22	0.51	0.84	0.67-1.04	0.112
SBP per 10 mmHg	1.2	1.07-1.35	0.002	1.1	0.95-1.26	0.213
DBP per 10 mmHg	1.23	1-1.51	0.049	0.95	0.74-1.22	0.691
Female (vs male)	0.69	0.43-1.12	0.132	0.96	0.54-1.68	0.875

Supplementary Table 4. Univariate and multi-variate Cox proportional hazards models for the risk of reaching end-stage kidney disease or having a sustained >30% decline in kidney function in all patients. eGFR: estimated glomerular filtration rate, ACR: urinary albumin:creatinine ratio, SBP: systolic blood pressure, DBP: diastolic blood pressure.

(A)

	Unadjusted, univariate analysis			Adjusted, multivariate analysis		
	**HR**	**95% CI**	**p**	**HR**	**95% CI**	**p**
log2 miR-190a-5p	0.61	0.45-0.82	0.001	0.58	0.4-0.84	0.004
Baseline eGFR per 10ml/min/1.73m^2	0.29	0.15-0.56	<0.001	0.22	0.08-0.6	0.003
ACR per 10 mg/mmol	1.08	1.03-1.14	0.003	1.06	0.99-1.14	0.094
Age per 10 years	1.19	0.87-1.64	0.282	0.56	0.33-0.93	0.026
SBP per 10 mmHg	1.07	0.83-1.39	0.597	1.02	0.76-1.37	0.915
DBP per 10 mmHg	1.16	0.73-1.83	0.54	1.16	0.63-2.15	0.64
Female (vs male)	0.45	0.16-1.3	0.142	0.87	0.21-3.59	0.842

(B)

	Unadjusted, univariate analysis			Adjusted, multivariate analysis		
	**HR**	**95% CI**	**p**	**HR**	**95% CI**	**p**
log2 miR-190a-5p	0.68	0.53-0.89	0.004	0.68	0.5-0.93	0.015
Baseline eGFR per 10ml/min/1.73m^2	0.36	0.22-0.59	<0.001	0.25	0.12-0.5	<0.001
ACR per 10 mg/mmol	1.02	1.01-1.03	0.002	1.02	1-1.05	0.049
Age per 10 years	1.28	0.96-1.71	0.09	0.61	0.39-0.95	0.027
SBP per 10 mmHg	1.21	0.99-1.47	0.058	1.02	0.8-1.3	0.884
DBP per 10 mmHg	1.28	0.9-1.83	0.166	1.05	0.64-1.73	0.842
Female (vs male)	0.54	0.22-1.35	0.188	0.97	0.33-2.89	0.96

Supplementary Table 5. Univariate and multi-variate Cox proportional hazards models for the secondary endpoint of progression to end-stage kidney disease in (A) patients with baseline urinary ACR <300 mg/mmol only and (B) all patients.

eGFR: estimated glomerular filtration rate, ACR: urinary albumin:creatinine ratio, SBP: systolic blood pressure, DBP: diastolic blood pressure

Mir-190 Predicted target	p_val of correlation
Trove2	5.10E-04
Epc2	6.85E-04
Kpna4	0.003041614
Nfib	0.003170286
Adam10	0.004121565
Ctdnep1	0.004193721
Sestd1	0.007890174
Krit1	0.009262762
Scyl3	0.009456387
Akirin1	0.009761816
Cramp1l	0.010293993
Asb3	0.011697136
Ttc5	0.012040002
Tmem216	0.013245681
Ctnnd1	0.013704996
Anxa7	0.014088805
Tbc1d15	0.014276163
Hmgb3	0.015780138
Tsc22d4	0.015855117
Serbp1	0.016467173
Lats2	0.017702381
Ppp4r2	0.018774895
Tagap1	0.019390408
Btg3	0.020493569
Brd1	0.021947848
Sugt1	0.022464252
Abhd6	0.025905883
Dynlt3	0.030046297
Ccser1	0.031692494
Mga	0.032611696
Msi2	0.033261378
Fam135a	0.035270094
Ccnt1	0.036591634
Fubp1	0.037596378
Rnf19a	0.037964711
Ahr	0.038438435
Akap9	0.038931266
Cask	0.041335262
Ptp4a1	0.042672374
Syncrip	0.043280774
Btbd10	0.045590656
Spry2	0.045728361
Sec63	0.046337383

Supplementary Table 6. List of predicted miR-190a-5p target genes for which the mRNA was highly negatively correlated with miR-190a-5p expression in the kidney cortex. 247 total miR-190a-5p targets were identified using only interactions which were conserved and present in more than one database. For determination of linear correlation between variables, correlation coefficients (r) were generated using Pearson's test. Genes were included if correlation significance was $p<0.05$.
