# Supplementary material for: Low circulating miR-190a-5p predicts progression of chronic kidney disease
Source: Nat Commun. 2025 Oct 15;16:9154. doi: 10.1038/s41467-025-64168-6 (PMC12528679; doi:10.1038/s41467-025-64168-6)
Supplement: Supplementary file 2 — Description Of Additional Supplementary File [file 41467_2025_64168_MOESM2_ESM.pdf]

Description of supplementary Additional files

Supplementary Data 1 Circulating MiRNome of discovery cohort.

Count table of mapped miRNAs. After count normalisation and removal of lowly expressed reads, differential expression analysis was performed using EdgeR. A fold change > 1.5 and false discovery rate (FDR, Benjamini-Hochberg method) < 0.05 was considered significant. Spreadsheet of normalised counts is in Supplemental data file.
